# Fatty Pancreas-Centered Metabolic Basis of Pancreatic Adenocarcinoma: From Obesity, Diabetes and Pancreatitis to Oncogenesis

**DOI:** 10.3390/biomedicines10030692

**Published:** 2022-03-17

**Authors:** Ming-Ling Chang

**Affiliations:** 1 Division of Hepatology, Department of Gastroenterology and Hepatology, Chang Gung Memorial Hospital, Taoyuan 333423, Taiwan; mlchang8210@gmail.com; Tel.: +886-3-3281200-8107; Fax: +886-3-3272236; 2 College of Medicine, Chang Gung University, Taoyuan 333323, Taiwan

**Keywords:** fatty pancreas, PDAC, metabolic, obesity, fatty infiltration, fatty replacement

## Abstract

Pancreatic ductal adenocarcinoma (PDAC) is one of the deadliest types of cancer, and it is currently the third most common cause of cancer death in the U.S.A. Progress in the fight against PDAC has been hampered by an inability to detect it early in the overwhelming majority of patients, and also by the reduced oxygen levels and nutrient perfusion caused by new matrix formation through the activation of stromal cells in the context of desmoplasia. One harbinger of PDAC is excess intrapancreatic fat deposition, namely, fatty pancreas, which specifically affects the tumor macro- and microenvironment in the organ. Over half of PDAC patients have diabetes mellitus (DM) at the time of diagnosis, and fatty pancreas is associated with subsequent DM development. Moreover, there is a strong association between fatty pancreas and fatty liver through obesity, and a higher intrapancreatic fat percentage has been noted in acute pancreatitis patients with DM than in those without DM. All these findings suggest that the link between fatty pancreas and PDAC might occur through metabolic alterations, either DM-related or non-DM-related. Based on clinical, in vivo and in vitro evidence, the current review highlights the etiologies of fatty pancreas (including fatty infiltration and replacement) and the fatty pancreas-associated metabolic alterations involved in oncogenesis to provide crucial targets to prevent, detect, and/or effectively treat PDAC.

## 1. Introduction

Pancreatic cancer is an umbrella term for several malignancies. Approximately 90% of these cancers are pancreatic ductal adenocarcinoma (PDAC) [1], among the deadliest types of cancer [2]. Currently, PDAC is the third most common cause of cancer death in the United States [3]. Estimates of PDAC incidence and mortality in the worldwide general population are 8/10^5^ person-years and 7/10^5^ person-years, respectively [2]. Moreover, progress in the fight against PDAC has been hampered by many factors, such as an inability to detect the disease early in the overwhelming majority of patients [4]. In addition, the reduced oxygen levels and nutrient perfusion caused by new matrix formation through activation of stromal cells in the context of desmoplasia are involved [5], as characterized by vascular deficiency and abundant desmoplastic stroma (which usually represents 90% of the tumor volume) [6]. It has been established that PDAC does not arise de novo but is preceded by noninvasive precursor lesions including pancreatic intraepithelial neoplasia (PanIN), intraductal papillary mucinous neoplasm (IPMN) and mucinous cystic neoplasm [6]; these lesions undergo histologic and genetic progression, culminating in invasive neoplasia [7]. Excess adiposity is considered causally related to PDAC [8], and one harbinger of PDAC is excess intrapancreatic fat deposition, namely, fatty pancreas, affecting the tumor macro- and microenvironment, specifically in the pancreas. The presence of IPMN [4] and PanIN lesions is also associated with fatty pancreas [9], which has been investigated in the setting of pancreatitis, a major risk factor for pancreatic cancer [4]. Nearly two-thirds of PDAC patients have diabetes mellitus (DM) at the time of diagnosis [7]; fatty pancreas is independently associated with subsequent DM development [10] (particularly among lean individuals) [11]; a strong association exists between fatty liver and fatty pancreas; and both are linked to obesity [12,13]. Indeed, a higher intrapancreatic fat percentage has been detected in acute pancreatitis patients with DM than in those without DM [14]. Taken together, findings to date suggest that the link between fatty pancreas and PDAC might be through metabolic alterations, either DM-related or non-DM-related. Based on clinical, in vivo and in vitro evidence, the current review aims to highlight the etiologies of fatty pancreas (including fatty infiltration and replacement) and fatty pancreas-associated metabolic mechanisms responsible for oncogenesis to contribute to preventing, detecting, and/or effectively treating PDAC.

## 2. Survey of Fatty Pancreas

Several protocols are used to measure pancreatic fat via magnetic resonance imaging (MRI), with MR spectroscopy (MRS) being considered equivalent to histology. Although histology remains the gold standard for assessing pancreatic fat content, routine biopsy sampling to evaluate fatty pancreas is not feasible [15].

With regard to histology, there is currently no validated objective scoring system available for the histologic assessment of fatty pancreas [16]. Based on 394 consecutive autopsies, Olsen et al. established a subjective grading score from 1 to 4, where 1 represents few scattered adipocytes in the exocrine pancreas and 4 represents partial or complete replacement by fat [17]. Moreover, fat accumulation in the pancreas may be even or uneven [18,19,20,21], and four types of uneven fatty pancreas have been described: (1) Type 1a, featuring replacement of the head with sparing of the uncinate process and peribiliary region; (2) Type 1b, characterized by replacement of the head, neck, and body, with sparing of the uncinate process and peribiliary region; (3) Type 2a, featuring replacement of the head, including the uncinate process, and sparing of the peribiliary region; and (4) Type 2b, characterized by the total replacement of the pancreas with sparing of the peribiliary region. Types 1a, 1b, 2a and 2b account for 35%, 35%, 12% and 18% of the cases with fatty pancreas, respectively [18,22].

For MRI, there are several methods to measure fatty pancreas [21], including spectroscopy (i.e., MRS) and imaging methods which focus on chemical-shift imaging and evolve into spectral-spatial fat-selective, multipoint Dixon and, ultimately, proton-density fat fraction (PDFF) techniques [23]. As mentioned, MRS is considered the gold standard for non-invasive pancreatic fat quantification. This technique requires the user to manually position a voxel to contain as much pancreatic tissue as possible [23]. Of note, the normal pancreatic fat cut-off of 6.2% is recommended for defining fatty pancreas [24].

## 3. Obesity, Fatty Pancreas and PDAC

Hypotheses to explain the association between obesity and pancreatic cancer risk include hormonal and inflammatory effects of adipose tissue, increased exposure to carcinogens, reduced physical activity, obesity-induced hypoxia resulting in increases in vascular endothelial growth factor [25] and an altered gut microbiota [26]. In obesity, lipid metabolism is influenced by ectopic fat depots, including fatty pancreas [27]. A wide range of terms have been applied to describe the phenomenon of pancreatic fat accumulation (Table 1). In particular, the incidence of non-alcoholic fatty pancreas disease (NAFPD) varies from 16% to 69.7%, depending on the country [28]. There are two forms of fatty pancreas: fatty infiltration and fatty replacement. The former is characterized by the accumulation of fat in the pancreas and is associated with metabolic syndrome and/or obesity, defining NAFPD; the latter is characterized by the death of pancreatic acinar cells and their replacement by adipocytes [29]. Risk factors for fatty replacement include congenital diseases (Shwachman–Diamond syndrome, Johanson-Blizzard syndrome, cystic fibrosis and heterozygous carboxyl-ester lipase mutation), alcohol abuse, infections, hemochromatosis, medicines and malnutrition [30]. Clinically, fatty pancreas is associated with various types of endocrine and exocrine dysfunction, such as type II DM (T2DM), cystic fibrosis, hemochromatosis [31], pancreatitis, premalignant lesions, pancreatic cancer [8], intraoperative blood loss [21] and postoperative pancreatic fistula [32,33]. The association of ectopic fat deposition in various tissues with tissue dysfunction and metabolic derangements is known as lipotoxicity [34]. In addition to lipotoxicity, the mechanisms by which fatty pancreas affects β-cell function are associated with islet inflammation or pancreatic innervation remodeling [35]. Additionally, the intracellular accumulation of nonesterified fatty acids (NEFAs) and triglycerides promotes mitochondrial uncoupling, oxidative stress, endoplasmic reticulum stress and altered membrane composition and function, ultimately promoting inflammation and cell death [27]. The effects of insulin and/or insulin-like growth factor-1 (IGF-1) are mediated via insulin receptor, IGF-1 receptor, and hybrid insulin and IGF-1 receptor binding, with the subsequent activation of the phosphoinositide 3-kinase signaling cascade. Notably, insulin and IGF-1 receptors are expressed on human pancreatic cancer cells [2]. Furthermore, the effects of insulin are mediated by insulin receptors and IGF-1 present on activated pancreatic stellate cells (PaSCs) and by the AKT/mammalian target of rapamycin (mTOR) downstream signaling. Through effects on PaSCs, obesity and T2DM contribute to pancreatic fibrogenesis and desmoplasia, promoting PDAC [2], and the pancreatic inflammatory process within the context of fatty pancreas is an important predisposing factor for the development of PDAC [36].

## 4. Factors Associated with Acinar-to-Adipocyte Transdifferentiation

Changes in cellular identity within the pancreas can be triggered by recurrent attacks of pancreatitis and low-grade inflammation. These changes may lead to pancreatic carcinogenesis [8], and acinar-to-ductal metaplasia (ADM) is an initial step of KRAS-driven pancreatic carcinogenesis [39]. Another change in the identity of acinar cells, namely, acinar-to-adipocyte transdifferentiation (AAT), also contributes to fatty pancreas [8]. Several intrinsic factors, such as the transcription factors c-Myc, GATA-binding factor 6 (GATA6), hepatocyte nuclear factor 6 (HNF6), liver kinase B1 (LKB1) and EWSR1-FLI1, and extrinsic factors including the extracellular matrix molecule periostin (Postn), are involved in AAT [8,39]. Specifically, the basic helix-loop-helix transcription factor c-Myc is one of the main regulators of AAT. Pancreatic growth in mice lacking c-Myc is impaired, and the loss of acinar cells increases over time, concomitantly with adipose tissue accumulation. Pancreatic adipose cells derive directly from transdifferentiating acinar cells [40]. Gata6 is expressed in all epithelial cells in the adult mouse pancreas, though it is only essential for exocrine pancreas homeostasis. A massive loss of acinar cells and fat replacement occurs after pancreas-selective Gata6 inactivation, which is accompanied by increased acinar apoptosis and proliferation, ADM and AAT [41]. Mice with postnatal duct-specific deletion of HNF6 or LKB1 (also known as serine/threonine kinase 11 (STK11)) exhibit pancreatic duct dilation, which is associated with chronic pancreatitis, including ADM, acinar proliferation and apoptosis, inflammatory infiltration, fibrosis and lipomatosis. The duct-specific inactivation of the above genes leads to AAT. Such injuries can account for the increased risk of developing pancreatic cancer in Peutz–Jeghers patients who harbor LKB1 loss-of-function mutations [42]. Ewing sarcoma depends on the occurrence of the EWSR1-FLI1 fusion oncogene, and the expression of EWSR1-FLI1 affects protumorigenic pathways and induces cell transformation. In particular, two conditional mouse models expressing mutant Kras^G12D^ (KC) or the EWSR1-FLI1 oncogene (E/F) in pancreas cells exhibited prominent acinar cell mass depletion and extensive lipomatosis. Moreover, E/F mice exhibit spontaneous ADM formation without the development of neoplastic lesions [43]. Postn is secreted by PaSCs and is crucial for proper exocrine lineage-specific regeneration after severe acute pancreatitis. Pancreatic stellate cells are the only source of Postn secretion in the human pancreas, and the loss of Postn expression is accompanied by strong pancreatic atrophy and AAT. An excessive desmoplastic reaction is also a typical characteristic of PDAC, in which Postn is strongly expressed [44].

## 5. Animal Studies of Fatty Pancreas

### 5.1. Fatty Pancreas in Obese Transgenic Mice

Compared to lean mice, the pancreas of leptin deficient (Lep^ob^) and obese hyperleptinemic (Lep^db^) mice has a higher total pancreatic fat, triglyceride and free fatty acid content but lower phospholipid and cholesterol content. Triglycerides and free fatty acids represent the toxic components of adipose tissue, and both phospholipids and cholesterol are integral constituents of cell membranes; thus, despite an overall increase in adiposity, the membrane stability and fluidity appear to remain constant in the case of fatty pancreas [45].

### 5.2. Fatty Pancreas in Diet-Induced Obese Mice/Rats

In mice exposed to high-fat diets (HFDs), the pancreas seems to be more susceptible to fat deposition than the liver; additionally, higher insulin resistance, increased pancreatic fat, increased inflammation, greater fibrosis [29], larger pancreatic islet size and greater α- and β-cell immunodensities are found than in mice fed standard chow [46]. Consistently, fat accumulates in pancreatic acinar cells with subsequent pancreatic fibrosis and acinar cell injury in Zucker diabetic fatty rats fed HFDs [47]. In addition, the increased fat storage and decreased pyruvate dehydrogenase complex activity in β-cells account for the abnormal insulin secretion of rats fed a sucrose-rich diet [48], and rosiglitazone has been shown to exacerbate fatty pancreas in high-fat/high-sucrose diet-fed mice [49]. Spleen-derived IL-10, a potent anti-inflammatory cytokine, may protect against the development of NAFPD, but obesity reduces IL-10 production in HFD-fed mice [50].

### 5.3. Fatty Pancreas in Non-Obese Cotton Rats

The islets of non-obese cotton rats fed a normal diet increase in size through β-cell hyperplasia with insulin resistance, and adipocytes accumulate in the pancreas but not in the liver during aging [51].

### 5.4. Fatty Pancreas in Offspring

Gestational DM in rats causes long-term effects on the pancreas of offspring, whereas n-3 polyunsaturated fatty acids have a beneficial role [52]. This is probably because maternal obesity alters endoplasmic reticulum homeostasis in the pancreas of offspring. In mice, regulators of kinase RNA-like ER kinase, inositol requiring 1 alpha protein, and activating transcription factor 6 pathways are affected by obesogenic insults [53]. Moreover, developmental programming participates in the pathogenesis of NAFPD and appears to be largely dependent on an adverse extrauterine environment [54]. Additionally, fetal and neonatal exposure to a maternal obesogenic environment interacts with a postnatal hypercaloric environment to induce offspring NAFPD through mechanisms involving perturbations in core circadian gene expression [55].

### 5.5. Fatty Pancreas and PDAC

In animal models, excess intrapancreatic fat is a driver of pancreatic carcinogenesis [56]. For example, pigment epithelium-derived factor (PEDF), a non-inhibitory serine protease inhibitor with potent antiangiogenic activity, is implicated in metabolism and adipogenesis. EL-Kras (G12D)/PEDF-deficient mice develop invasive PDAC associated with enhanced matrix metalloproteinase expression, increased peripancreatic fat, adipocyte hypertrophy and intrapancreatic adipocyte infiltration. These data suggest that an adipose-rich environment may drive tumor growth and progression [57].

## 6. Human Studies of Fatty Pancreas

Throughout life, there is important interplay between the endocrine and exocrine pancreas [34]. Below, we summarize the findings of human fatty pancreas studies regarding the exocrine and endocrine functions of the pancreas, pancreatitis, premalignancy and PDAC.

### 6.1. Fatty Pancreas and Pancreas Exocrine Function

Three mechanisms have been proposed to induce fatty pancreas-associated exocrine pancreatic disorder: the lipotoxicity of acinar cells, adipocyte-mediated negative paracrine effect, and direct destruction of acinar cells [36]. A study of 1458 volunteers who underwent MRI of the pancreas reported an inverse correlation between the PDFF of the pancreas and fecal elastase, suggesting an association between fatty pancreas and impaired pancreatic exocrine function [58]. However, another study involving 109 participants with a glucagon stimulation test and N-benzoyl-L-tyros-p-amino benzoic acid as well as unenhanced abdominal computed tomography (CT) found no clear relationship between fatty pancreas and pancreatic exocrine impairment [59]

### 6.2. Fatty Pancreas and Metabolic Profiles

Overall, it remains debated whether fatty pancreas is associated with glucose metabolism, including DM, insulin resistance, β-cell function, prediabetes, fasting glucose, glucose tolerance, glycated hemoglobin, T2DM risk, metabolic syndrome and a longitudinal decrease in endogenous insulin-secreting capacity. For example, positive associations were noted in a meta-analysis of 13 studies involving 49,329 subjects [60], a meta-analysis of 17 studies with 11,967 individuals [61], a cross-sectional study of 4419 Chinese individuals [62], and a pooled analysis of 12,675 individuals [24]. Positive correlations were also noted in studies involving the following participants: 121 consecutive children with echographic evidence of fatty liver [63]; 25 lean adolescents and 24 adolescents with obesity using MRI [64]; 198 participants without diabetes [65]; 78 Chinese T2DM subjects and 35 volunteers without diabetes [66]; 50 prepubertal children with obesity and 30 children with a lean status via ultrasonographic assessment [67]; 97 sedentary 40–55-year-old individuals [68]; 361 Caucasians at an increased risk of T2DM [69]; 105 patients with iron overload [70]; 45 patients with T2DM and 81 “at risk for T2DM” [71]; 56 patients with T2DM [35]; 65 children with NAFLD [72]; 39 Korean participants without a previous history of diabetes who underwent 1H-MRS [73]; 310 individuals with a body mass index (BMI) ≥ 25 kg/m^2^ and serum triglycerides ≥ 1.7 mmol/l and/or T2DM [74]; 106 subjects at T2DM onset [75]; 14 T2DM patients, 13 age- and sex-matched healthy controls (HCs) and 11 young HCs using 3T Prisma MRI [76]; 132 consecutive T2DM patients [77]; 143 patients with NAFLD [78]; 79 patients undergoing total pancreatectomy with islet autotransplantation [79]; 27 subjects with morbid obesity and 15 HC subjects with bariatric surgery [80]; 50 age- and BMI-matched normal subjects, 51 with newly diagnosed type 2 diabetes (T2D-new), 53 with T2D < 5 years (T2D < 5Y), and 52 with T2D ≥ 5 years (T2D ≥ 5Y) [81]; 685 healthy volunteers with fat-water MRI and H-MRS [82]; 250 consecutive patients with endoscopic ultrasonography examination [83]; and 320 participants [84], 112 volunteers [85], 109 participants [59], 64 patients [86], 51 subjects [87], 45 subjects [88], and 78 individuals [89] without obesity. Moreover, as short-term training efficiently reduces ectopic fat within the pancreas, exercise training may reduce the risk of T2DM [68]. Conversely, negligible associations between fatty pancreas and metabolic profiles were found in the following studies: a meta-regression analysis of 9 studies with 1209 healthy individuals [24]; a randomized controlled trial of 137 adults with obesity but not diabetes and weight loss [90], and studies of 277 eligible participants with a long-term low-fat or Mediterranean/low-carbohydrate diet [91], 73 persons without diabetes [92], 158 children and adolescents with overweight/obesity [93], 8 monozygotic male twin pairs [94], 86 patients [95], pancreas sections of 72 Japanese nondiabetic (NDM) autopsy cases and 50 patients with diabetes and 49 age- and BMI-matched NDM patients who underwent pancreatic surgery [96], 1367 volunteers who underwent whole-body MRI and proton density fat fraction [97], 56 participants who underwent a frequent-sampling oral glucose tolerance test and MRI and 1H-MRS [98], and 97 women 3 to 16 months after pregnancy [99]. Theoretically, pancreatic fat accumulation may impair β-cell function via mechanisms including local free fatty acid release, triglyceride metabolite accumulation, oxidative stress, release of proinflammatory and vasoactive factors [100], apoptosis and subsequent fatty replacement [101], all of which induce β-cell injury. Nevertheless, the baseline demographic and glucose metabolic characteristics and methods used to investigate pancreatic fat in the aforementioned studies vary, and there are ethical differences regarding adipocyte size, ectopic fat deposition and diabetes predisposition [21,95,102,103,104,105]. The effect of fatty pancreas on insulin secretion appears to be highly dependent on genetic predilection to diabetes and other metabolic profiles [106,107,108], and there are various inflammation severities of islets [106]. All of these factors might account for the conflicting results. Moreover, metabolic status persistently affects the differentiation and lipolysis of pancreatic adipocytes, as shown by the defective upregulation of the genes governing adipogenesis, lipogenesis, and lipolysis during the differentiation of cells from T2DM patients [109]. Notably, triglycerides, glycated hemoglobin [61,81], T2DM, C18:1n-9 (oleic acid), uric acid, plasminogen activator inhibitor-1 [88], central obesity, hyperferritinemia [82] and lipocalin-2 [110] are factors associated with fatty pancreas. For women, aging and menopause are also related to fatty pancreas [111]. In patients with a body mass index ≥ 25 kg/m^2^ and serum triglycerides ≥ 1.7 mmol/l and/or T2DM, pancreatic fat is positively associated with only two metabolites: lysine-derivate and the bile acid conjugate taurodeoxycholate [74]. In patients with NAFLD, serum amylase and lipase levels and mesenteric fat correlate with the presence of severe fatty pancreas [78].

### 6.3. Fatty Pancreas and Pancreatitis

Following acute pancreatitis, levels of triglycerides [112], leptin and tumor necrosis factor-α (TNFα) [113] and biliary origin are associated with fatty pancreas [114]. In particular, the Raynaud index is the best biomarker of fatty pancreas in individuals with new-onset prediabetes or diabetes after acute pancreatitis [114]. A higher intrapancreatic fat percentage has been detected in acute pancreatitis patients with DM than in those without DM [14].

### 6.4. Fatty Pancreas and Fatty Liver

The arbitrary threshold for considering fatty liver as a histology feature is the presence of lipid droplets in >5% of hepatocytes [115]. The phenotypic spectrum of non-alcoholic fatty liver disease (NAFLD), defined as a fatty liver not associated with alcohol consumption, spans from simple steatosis to non-alcoholic steatohepatitis (NASH), which may lead to hepatocellular carcinoma (HCC). The precise event cascade leading from NAFLD progression to HCC is intricate and might entail diverse triggers, encompassing an altered immune response, oxidative and endoplasmic reticulum stress, organelle derangement, DNA aberrancies [116] and autophagy [53]. Similarly, NAFPD may lead to non-alcoholic steatopancreatitis (NASP), and both NAFPD and NASP can promote the development of pancreatic cancer [117]. Given that total pancreatic fat is a predictor for the presence of NAFLD, a strong association exists between NAFPD and NAFLD, and that both are linked to obesity [12,13], it is likely that NAFPD shares similar pathogenic mechanisms with NAFLD to induce PDAC [53]. We thus compared the characteristics of fatty liver and fatty pancreas. As shown in Table 2, the liver and pancreas originate from the same embryonic endoderm [118] and share the same vagal motor neuron origin, namely, the amygdala [119], and both fatty pancreas and fatty liver increase posttransplant complications [120,121]. However, the pancreas seems to be more susceptible to fat deposition than the liver [122], as a total of 68% of subjects with fatty pancreas have fatty liver, but up to 97% of subjects with fatty liver have fatty pancreas [21]; fatty pancreas is even considered the first site of ectopic lipid deposition [15]. Thus, NAFPD was shown to precede the development of NAFLD and may serve as a metabolic risk marker [123]. Unlike fatty liver, in which fat accumulates in hepatocytes, triglycerides mainly accumulate in pancreatic tissue adipocytes by “fatty infiltration” of the pancreas [122]. Among NAFLD patients, 10.3% have a high risk of advanced fibrosis, but NAFPD may or may not be associated with advanced fibrosis or chronic pancreatitis [83,124,125,126]. Transient elastography, such as FibroScan, is a good noninvasive test for assessing fibrosis in NAFLD but not in NAFPD [12]. Although the relationship between fatty pancreas and glucose metabolism remains a subject of ongoing investigation, liver fat volume fractions correlate more closely than pancreatic fat volume fractions with insulin resistance and β-cell function [127]. Furthermore, the liver is an organ that takes up, oxidizes, synthesizes and exports fatty acids, whereas the pancreas does not have those functions [35]. After correcting for BMI, the association between fatty liver and fatty pancreas vanishes, indicating that their association occurs through obesity [21]. Interestingly, despite the association between NAFLD and NAFPD, fat loss in the liver and pancreas occurs independently after patients undergo bariatric surgery, suggesting tissue-specific mobilization of ectopic fat deposits [21,88]. Weight loss may lead to a more rapid decrease in triglycerides in pancreatic and hepatic cells than in adipocytes between pancreatic cells [90], and pancreatic fat loss is mainly associated with improved lipid, rather than glycemic, profiles [91]. In general, the interplay between fatty pancreas and fatty liver is complex. For example, a study of 50 children showed that fatty pancreas and fatty liver have complementary clinical consequences: fatty liver demonstrated a dominant effect, and even relatively small degrees of fatty pancreas may contribute to metabolic alterations [100]. In addition, pancreatic fat accumulation may play a pivotal role during the intermediary step of the disease and negatively affect insulin secretion only via persistent insulin resistance, when β-cells can no longer compensate for the increased insulin demand [105]. The presence of fatty pancreas is related to the aggravation of the NAFLD disease severity [30] in terms of liver fibrosis, ballooning and NAFLD activity scores [63]. Intriguingly, a cross-sectional study of 43 adult patients with biopsy-proven NAFLD showed that increased pancreatic fat is associated with fatty liver, but that liver fibrosis is inversely associated with pancreatic fat content [128]. The sophisticated interplay between the liver and pancreas might occur through, at least partly, fetuin-A, a hepatokine released from a fatty liver. Furthermore, fetuin-A-mediated metabolic crosstalk of a fatty liver with islets may contribute to an obesity-linked glucose blindness of β-cells, and fatty pancreas may exacerbate local inflammation [108,129]. Fat cells from the perivascular tissue of the pancreas appear to be particularly responsive to combined signals of saturated fatty acids and fetuin-A [130].

### 6.5. Fatty Pancreas and Pancreatic Cancer

The links between fatty pancreas and PanIN [9], IPMN [4,131], early-stage PDAC [132], PDAC [4,8,133] and lymphatic metastases in PDAC [134,135] have been well established. Moreover, several studies have shown that fatty pancreas is associated with pancreatic cancer after accounting for waist circumference or BMI, which suggests the existence of fatty pancreas-specific pathways other than the overflow of fat into the pancreas from the visceral fat depot [8]. We want to stress again that the mechanism by which fatty pancreas contributes to pancreatic cancer is primarily related to adipose tissue inflammation, including the release of proinflammatory adipokines [136] and cryptogenic pancreatic inflammation with fatty changes [137], similar to what occurs in NAFLD [138]. In the case of fatty infiltration, such as NAFPD, fat accumulates in the pancreas of individuals without pancreatic disease; in contrast, fatty replacement is mainly found in individuals with pancreatic diseases such as pancreatitis. Interestingly, both fatty infiltration and fatty replacement might coexist and contribute simultaneously to PDAC. Specifically, obesity/diabetes/insulin-related pathways and pancreatic cell injury pathways might be responsible for pancreatic carcinogenesis in fatty infiltration and fatty replacement, respectively. In patients with T2DM or those with chronic pancreatitis [139], some β-cells lose their identity and regress to a precursor-like dedifferentiated state. Both ADM [140] and AAT [8,39,40,41,42,43,44] might contribute to fatty replacement of the pancreas and subsequent carcinogenesis [8]. Additionally, persistent organic pollutants (POPs) are lipophilic toxins which are able to bioaccumulate in fatty-rich tissues of animals [21], and a fatty pancreas creates a neurotrophic microenvironment and promotes the remodeling of pancreatic innervation [141]. Both POPs and pancreatic innervation might contribute to the development of PDAC.

## 7. Reprogrammed Metabolism of PDAC

Reprogrammed metabolism and metabolic crosstalk within the tumor microenvironment contribute to unlimited pancreatic tumor proliferation [142]. An early event during malignant transformation is the acquisition of activating mutations in the KRAS oncogene, occurring in >90% of PDAC patients, and such mutation is a major contributor to PDAC initiation, progression and metastasis [1]. Oncogenic KRAS promotes metabolic rewiring to support increased biosynthetic demand by reprogramming glucose and glutamine (Gln) metabolism as well as increasing autophagy and macropinocytosis. [5]. These metabolic alterations can also promote PDAC progression through epigenetic regulation and are closely associated with chemoresistance, radioresistance and immunosuppression [142].

The reprogrammed metabolism of PDAC is summarized below.

### 7.1. Aerobic Glycolysis and Noncanonical Gln Metabolism

Otto Warburg demonstrated that tumor cells consume more glucose than normal cells. Tumor cells subsequently convert most glucose-derived carbon into lactate, even in the presence of sufficient oxygen, a process known as the Warburg effect or aerobic glycolysis [143]. In PDAC, KRAS upregulates several key enzymes for glycolytic processes, including glucose transporter 1, hexokinase 1, hexokinase 2, phosphofructokinase 1 and lactate dehydrogenase A [144]. Furthermore, mitochondrial oxidative phosphorylation is suppressed in pancreatic cancer cells [145]. In the canonical pathway, Gln enters the mitochondria and is converted to glutamate, and then to α-ketoglutarate by the enzyme glutamate dehydrogenase 1 (GLUD1). Oncogenic KRAS downregulates GLUD1 and upregulates aspartate transaminase (GOT1) to generate pyruvate and nicotinamide adenine dinucleotide phosphate [5]. This process is termed KRAS-driven noncanonical Gln metabolism [146].

### 7.2. Lipid Metabolism

In contrast to normal cells relying on dietary fat, >90% of triacylglycerol fatty acids in tumor cells are synthesized de novo [147]. Thus, in PDAC, the enzymes participating in de novo fatty acid and cholesterol synthesis, including ATP citrate lyase, citrate synthase, stearoyl-CoA desaturase, fatty acid synthase and 3-hydroxy-3-methylglutaryl coenzyme A reductase [148], are all upregulated. In addition, hypoxia or oncogenic KRAS accelerates monounsaturated fatty acid uptake from extracellular lysophospholipids [149], and overactive low-density lipoprotein receptor-mediated uptake of cholesterol-rich lipoproteins is noted in murine pancreatic cancer cells [148]. Pancreatic cancer cells are able to inhibit Gln degradation in cocultured adipocytes and render them able to secrete Gln, and the Gln derived from adipocytes augments cancer cell proliferation [150]. Additionally, adipocyte accumulation might interact with pancreatic stellate cells and tumor-associated neutrophils to enhance tumor progression, particularly in obese patients [151].

### 7.3. Autophagy and Macropinocytosis

Autophagy and macropinocytosis are critical nutrient supply pathways that support the high rate of proliferation for pancreatic tumor growth in nutrient-deprived environments [152]. Autophagy occurs through the inhibition of the mTOR pathway [152], and the MAPK pathway is involved in macropinocytosis induction [5].

**Figure 1:**
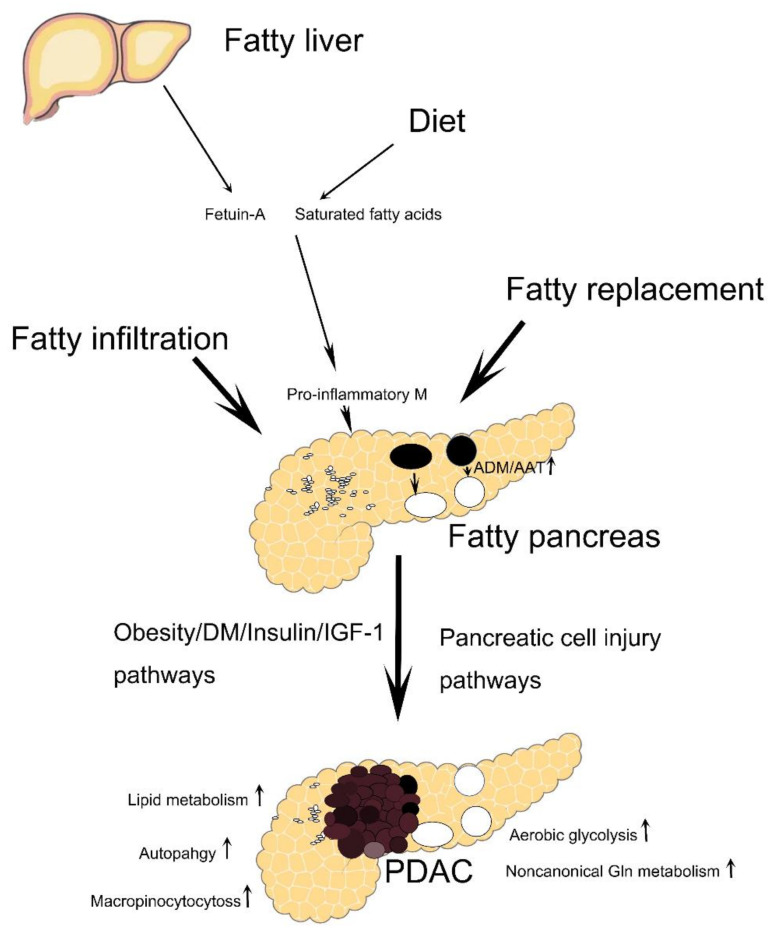
The proposed link between fatty pancreas and pancreatic ductal adenocarcinoma (PDAC). Fatty liver-derived fetuin-A and saturated fatty acids such as palmitate stimulate pancreatic fat cells and islet resident macrophages. Specifically, both fetuin-A and palmitate augment toll-like receptor 4 expression in pancreatic adipocytes, which in turn increase the secretion of interleukin-6, interleukin-8 and monocyte chemoattractant protein-1 and stimulate pancreatic resting macrophages to become pro-inflammatory macrophages [129]. The pro-inflammatory macrophages and the associated inflammatory milieu might lead to PDAC, as the pancreatic inflammatory process within the context of fatty pancreas is crucial for the development of PDAC [37]. Please see text for other details. ADM: acinar-to-ductal metaplasia; AAT: acinar-to-adipocyte transdifferentiation; M: macrophage; IGF-1: insulin-like growth factor-1; Gln: glutamine.

Figure 1 summarizes the connection between fatty pancreas and PDAC from the viewpoints of fatty infiltration and replacement, the interaction between fatty liver and fatty pancreas, ADM, AAT and metabolic reprogramming of PDAC.

## 8. Conclusions and Outlook

PDAC has the highest case fatality rate of any solid tumor, highlighting the urgency for novel therapeutic strategies to combat this deadly disease. Currently, targeted or immunotherapy strategies effective for most patients with PDAC are lacking. Accumulating animal and human evidence suggests that fatty pancreas is involved in PDAC development. Both fatty infiltration and fatty replacement lead to fatty pancreas and may predispose toward the development of PDAC in different or complementary ways. Further investigations to clarify the crucial factors that cause fatty pancreas, particularly those associated with metabolic oncogenesis, are needed. Such research is vital for discovering effective therapeutic strategies to combat PDAC by preventing the development of fatty pancreas.

## Figures and Tables

**Table 1:** Nomenclature and definitions of various forms of fatty pancreas [37,38].

Nomenclature	Definition
IPFD Pancreatic lipomatosis Pancreatic steatosis Fatty pancreas	General terms that can be used for all forms of pancreatic fat accumulation.
Fatty replacement	Damage of pancreatic acinar cells leading to their death, which then results in their replacement in the pancreas by adipocytes.
Fatty infiltration	Pancreatic infiltration of adipocytes caused by obesity.
NAFPD	Pancreatic fat accumulation in association with obesity and metabolic syndrome.
NASP	Pancreatitis owing to pancreatic fat accumulation.

IPFD: Intra-pancreatic fat deposition; NAFPD: Non-alcoholic fatty pancreas disease; NASP: Non-alcoholic fatty steatopancreatitis.

**Table 2:** Comparisons between fatty liver and fatty pancreas.

	Similarities	Differences	Characteristics
**Embryology**	Endoderm [118]		
**Vagal motor neuron origin**	Amygdala [119]		
**Post-transplant/post-operation complications.**	Increase [120,121]		
**Fat deposition**			Pancreas seems to be more susceptible to fat deposition compared with the liver [122]; 68% subjects with fatty pancreas harbored fatty liver, while up to 97% subjects with fatty liver had fatty pancreas [21]; Fatty pancreas is even considered the first site of ectopic lipid deposition [15]; NAFPD was shown to precede the development of NAFLD, and may serve as a metabolic risk marker [123].
**Histology**		Fatty liver: triglycerides in hepatocytes; Fatty pancreas: triglycerides in adipocytes [122].	
**Fibrosis**		10.3% of NAFLD patients: advanced fibrosis; NAFPD: may or may not associated [18,123,124,125]; Transient elastography; is a good noninvasive test to assess fibrosis in NAFLD but not for NAFPD [12].	
**Metabolism**		Liver is an organ that takes up, oxidizes, synthesizes and exports fatty acids. The pancreas does not have those functions [35].	Liver fat volume fractions > pancreatic fat volume to correlate with insulin resistance and β-cell function [127].
**Proposed progression**	NAFL → NASH → HCC; NAFP → NASP→ PDAC [21].		
**s/p corrected for BMI**		Association between fatty liver and fatty pancreas vanished [21].	
**s/p bariatric surgery**		Fat loss in the liver and the pancreas seem to be independent [21,87].	
**Weight loss**			More rapid decrease in triglycerides inside pancreatic and hepatic cells than inside adipocytes between pancreatic cells [90]; Pancreatic fat loss is mainly associated with improved lipid, rather than glycemic profiles [91].

NAFL: non-alcoholic fatty liver; NASH: non-alcoholic steatohepatitis; HCC: hepatocellular carcinoma; NAFP: non-alcoholic fatty pancreas; NASP: non-alcoholic steatopancreatitis; PDAC: pancreatic ductal adenocarcinoma.

## Data Availability

The datasets used and/or analyzed during the current study are available from the corresponding author on reasonable request.
